# Wild pistachio (*Pistacia atlantica mutica*) oil improve metabolic syndrome features in rats with high fructose ingestion

**DOI:** 10.22038/ijbms.2018.30511.7351

**Published:** 2018-12

**Authors:** Sanaz Jamshidi, Najmeh Hejazi, Mohammad-Taghi Golmakani, Nader Tanideh

**Affiliations:** 1 Department of Clinical Nutrition, School of Nutrition and food sciences, Shiraz University of Medical Sciences, Shiraz, Iran; 2 Nutrition Research Center, Department of Clinical Nutrition, School of Nutrition and Food Sciences, Shiraz University of Medical Sciences, Shiraz, Iran; 3 Department of Food Science and Technology, School of Agriculture, Shiraz University, Shiraz, Iran; 4 Stem Cell and Transgenic Research Center, Shiraz University of Medical Sciences, Shiraz, Iran

**Keywords:** Insulin resistance, Inflammation, Lipidemia, Metabolic syndrome, Oil, Pistachio, Pistacia atlantica

## Abstract

**Objective(s):**

Metabolic syndrome is a multiplex risk factor for diabetes and cardiovascular disease. Since some dietary fats such as mono-unsaturated fatty acids (MUFA) modify metabolic syndrome components the aim of the present study was to evaluate the preventive effects of mixture, kernel and hull oils of wild pistachio (WP) (*Pistacia atlantica mutica*) as good sources of MUFA on different features of this abnormality in rats under induction.

**Materials and Methods:**

In this study rats were randomly assigned to six groups with 12 animals per group. Metabolic syndrome was induced by fructose solution in groups 2, 3, 4, 5, and 6. Group 3 received sunflower oil and groups 4, 5, and 6 received mixture, hull and kernel oils of WP (2 ml/kg/day), respectively, for 10 weeks. Then, lipid profiles, glycemic indices, oxidative stress and inflammatory parameters were measured using standard laboratory tests.

**Results:**

Different forms of WP oil induced hypotriglyceridemia, but the hypocholesterolemia effect was seen only in the mixed and kernel oil groups. Kernel oil also significantly reduced LDL and HDL cholesterol (*P*<0.05). In addition, mixed and kernel oils notably decreased glycemic indices (fasting blood glucose and insulin resistance) compared with the fructose group. Serum insulin levels were significantly increased in the kernel oil group (*P*<0.05). All WP oils also significantly decreased inflammation (IL-6).

**Conclusion:**

The results showed that the consumption of WP kernel oil may have beneficial effects on preventing hyperglycemia, hypertriglyceridemia, hypercholesterolemia, inflammation and pancreatic secretory disorders.

## Introduction

Metabolic syndrome is a cluster of metabolic abnormalities that increase the risk of cardiovascular disease (CVD), diabetes, and so on (1, 2). These abnormalities include hyperglycemia, hypertriglyceridemia, low high density lipoprotein cholesterol (HDL-C) level, hypertension, and abdominal obesity (3). Due to increased obesity and sedentary lifestyle, the prevalence of metabolic syndrome has also increased and become an important public health problem all over the world (4).

Although the exact mechanism underlying metabolic syndrome is unclear, insulin resistance (IR) is known as its probable cause (5). In addition, oxidative damage due to imbalance between the antioxidant defense system and free radicals production has also been reported in metabolic syndrome patients (6).

At present, the main strategy for the management of metabolic syndrome is lifestyle modification including diet therapy. Dietary fat and antioxidants are two dietary components that affect the constituents of metabolic syndrome (7, 8).

Mediterranean dietary pattern, in which olive oil is the main source of fat, is confirmed as a primary and secondary preventive method of metabolic syndrome and its components (9). In this regard, monounsaturated fatty acids (MUFA) and antioxidants including phenolic compounds are effective (2, 8).

Therefore, the entry of local food sources containing MUFA and antioxidants into dietary pattern of people in each region may have preventive effects; one of these sources is pistachio family. *Pistacia atlantica mutica* or Baneh is a species of pistachio, which is native to a part of Euracia to north Africa (10). Zagros area of Iran is one of the regions with high dispersion of this wild pistachio (WP) (11). WP fruit contain dark green hull, wooden hard shell and a kernel which comprise 24, 51 and 25 percent of whole fruit respectively (12). Previous studies show beneficial effects of different parts of WP on features of diabete, dyslipidemia, inflammatory disorders, dental disease and peptic ulcer based on anti-microbial and antioxidation activities (13-16).

Fatty acid analysis of WP oil shows that it is a main source of oleic acid as WP hull and kernel oil is classified as a good source of MUFA, comprising of 66 and 54 percent MUFA while compared to olive oil it has about 62 percent (12, 17). Furthermore, these oils have notable content of natural phenolic antioxidants and other bioactive components such as beta-sitosterol even higher than other vegetable oils (12, 17).

However, to the best of our knowledge, there is no any study to evaluate the influence of these oils on metabolic syndrome risk factors, so in this study we evaluated the probable preventive effects of hull and kernel oils of WP on some components of metabolic syndrome. 

## Materials and Methods

***Oil preparation***

WP fresh fruits were collected in October 2016 from Nurabad (Zagros forest), Fars province, IRAN. The plant was identified by Mrs Sadigheh Khademian (Department of Traditional Pharmacy, Shiraz University of Medical Sciences, Shiraz, Iran) and its voucher herbarium specimen (no 2817) is deposited at the Herbarium of Shiraz Pharmacy School, IRAN.

The green hull and the kernel parts of fruits were separated and dried in the shade. Oil was obtained from these parts by cold press method (cold press device: calibre 35 mm, Iran) (17). Then, it was filtered and centrifuged at 4000 rpm during 15 min (Centrifuge machine, Sigma, Germany). Hull oil, kernel oil and mixture oil (60 percent hull and 40 percent kernel oils) were stored in dark bottles at 4 ^°^C for a maximum of one week. 

***Chemical analysis of the oil***

The fatty acid composition and sterol content were analyzed by gas chromatograph machine. Gas chromatography was performed on the samples, using a Beifen system (3420A, China) with a split/splitless injector, a flame ionization detector and a BPX70 capillary column (Bis-cyanopropylsiloxane-silphenylene, 30 m×0.25 mm internal diameter with 0.25 μm film thickness). The samples were injected into the column with the volume of 1 μl (split ratio of 1:10). Fatty acids were identified according to their retention times in comparison with the standards. Quantitative determination of fatty acids was carried out by calculating their relative peak areas. The sterol content was calculated according to National Iranian Standard No. ISIRI 9670, ISIRI 6081.

 The spectophotometric method of the International Dairy Federation was used to determine the peroxide value. The acid value (AV) was determined according to the AOCS Official Method Cd 3d-63. The amounts of phenols were estimated using the Folin-Ciocalteu reagent at 725 nm. Results were expressed as mg of gallic acid per g of samples.

Tocopherols were measured by RP-HPLC column VIT F (Knauer Smart Line, Germany) and UV-detection at 295 nm. Extracted oil was diluted with ethanol and 20 μl was injected to HPLC after filtration by a syringe filter (0.45 μ) with a C-18 lichrospher RP-100 (125 mm × 3 mm, 5 μm) column. The mobile phase was acetonitrile and water (80, 20 v/v) at a flow rate of 0.35 ml/min at 30 ^°^C temperature. Total tocopherol amount were calculated by comparing with standards purchased from Sigma.

***Experimental animals***

Seventy two male Sprague Dawley rats 6 to 8 week old (weighing 170-220 g each) were purchased from the center of experimental and comparative medicine, Shiraz University of Medical Sciences, Shiraz, Iran. The rats were housed in standard cages and maintained under controlled environment (temperature: 22±2^ °^C, lighting: 12 hr light/dark cycles and humidity: %50±5). The rats were fed a chow diet (Pars Dam Co, Tehran, IRAN) and also accessed to *ad libitum* drinking water. This study protocol was approved by the Ethics Committee of Shiraz University of Medical Sciences and was performed in accordance with the Ethical Standards laid down in the 1964 Declaration of Helsinki and its later amendments. 

***Induction of metabolic syndrome***

Metabolic syndrome was induced in Sprague Dawley rats through administering 8 g/kg body weight per day fructose by gavage (18). Fructose (Merch Co, Germany) solution was prepared at 50 percent concentration (as 50/50 with water).

***Experimental design***

The rats were divided randomly into six groups of 12 rats per group. 

Group 1, the control group, did not undergo induction of metabolic syndrome and received 2 ml normal saline as placebo for fructose solution and 0.5 ml normal saline twice per day for 10 weeks as oils placebo.

Group 2 received 2 ml fructose solution and 0.5 ml normal saline twice per day for 10 weeks.

Group 3 received 2 ml fructose solution and 0.5 ml sunflower oil for 10 weeks.

Group 4 received 2 ml fructose solution and 0.5 ml mixture oil of WP for 10 weeks.

Group 5 received 2 ml fructose solution and 0.5 ml hull oil of WP for 10 weeks.

Group 6 received 2 ml fructose solution and 0.5 ml kernel oil of WP for 10 weeks.

***Determination of biochemical parameters***

Rats were monitored weekly by weighing. At the end of week 10, after 12 hr fasting and under anesthesia (50 mg/kg ketamine plus 5 mg/kg diazepam through intra peritoneally administration), 5 ml blood was collected by cardiac puncture. The blood samples were centrifuged at 3500 g/min for 10 min to separate the serums and stored at -80 ^°^C prior to biochemical measurements. The lipid profile including triglyceride (TG), total cholesterol (TC), low density lipoprotein cholesterol (LDL-Chol), high density lipoprotein cholesterol (HDL Chol) and fasting blood glucose (FBG) was assayed by specific enzymatic kits (Pars Azmoon., Tehran, IRAN) (Autoanalyzer BT 1500, Medsystem, USA). Serum malondealdehyde (MDA) concentrations were determined by measuring TBARS (thiobarbituric acid reactive substances) by using spectophotometric assay (Spectrophotometry, Apel, Japan) (19). Serum concentrations of insulin and interlukin-6 (IL-6) were measured using available ELIZA kits (Monobind Inc, USA and IBL international, Germany, respectively) and superoxide dismutase (SOD) activity by Zellbio kit, Germany (Elisa microplate reader, Statfax 2600, USA). In addition, insulin resistance was measured by homestatic model assessment (HOMA -IR) based on this equation: fasting glucose (mmol/l)×fasting insulin (µIU/ml)/22.5 (20).

***Statistical analysis***

Statistical analysis was done by SPSS software, version 24 (SPSS Inc, Chicago IL). Normally distributed data were expressed as mean ± SD and abnormal data presented as median (IQR). Normally distributed data were compared between groups by one way ANOVA test (and TUKEY test as *post hoc*) and skewed data were compared by kruskal-wallis test (and Mann-witney U-test as *post hoc*). Repeated measurement test was also used to compare weight changes during the study weeks. A *P*-value of ≤0.05 was considered statistically significant.

## Results

***Chemical analysis of the oils***

Based on Table 1 major fatty acid in WPK (wild pistachio kernel) and WPH (wild pistachio hull) oils was oleic acid with 51.17 and 54.33 percentages but oleic acid contend of sunflower oil was 25 percent. In addition WPK oil has higher sterol content and beta sitosterol is the predominant phytosterol of WPK and WPH oils (84% and 82.44%), while beta sitosterol of sunflower oil was 61 percent. This study showed that WPH oil has higher peroxide value in comparison to WPK and sunflower oils (4.2, 0.8 and 0.7). Results showed higher content of total tochopherol in WPH oil (1070.49 mg/kg) compare to WPK oil (350.15 mg/kg) and sunflower oil (582 mg/kg).

After 10 weeks of consumption of fructose solution and different oils, the following results were obtained:

***Serum lipids***

The effects of interventions on lipid profile are shown in Table 2. There was a significant difference in TG, T-Chol, LDL-Chol and HDL-Chol between the groups (*P*-value =0.000). TG, T-Chol, LDL and HDL concentrations in the fructose group were significantly increased compared to the control group. 

Concentration of TG was significantly decreased in the mixture, WPH, WPK and sunflower oil groups compared to the fructose group (*P*-value =0.000) for each comparison, with maximum reduction in the mixture oil group. The mixture and WPK oils had a tendency to produce lower T-Chol concentrations compared to the fructose group (*P*-value =0.01 and 0.002, respectively). WPK oil also significantly reduced LDL-Chol and HDL-Chol compared to the fructose group (*P*-value = 0.002 and 0.02, respectively) and sunflower oil decreased LDL-Chol significantly compare to fructose group (*P*-value =0.001). In addition, HDL-Chol concentration significantly increased in the mixture and WPH oil groups compared to the control group (*P*-value =0.006, *P*-value =0.000).

***Glycemic indices***

Table 2 shows a significant difference between FBG, Insulin and HOMA-IR in 6 groups of the study (*P*-value =0.000 for FBG and Insulin and *P*-value =0.04 for HOMA-IR).This study showed a significant increase in FBG concentration and HOMA-IR in the fructose group compared to the control group (*P*-value =0.001, *P*-value =0.008). Mixture and WPK oils consumption significantly decreased the FBG (*P*-value =0.002, *P*-value =0.005) and HOMA-IR (*P*-value =0.007, *P*-value =0.024) compared to the fructose group. WPK oil consumption also increased the insulin level significantly (*P*-value =0.04).

FBG and insulin serumic concentrations were significantly lower and higher respectively in the groups which consumed mixture and WPK oils compared to sunflower oil (*P*-value =0.000).

***Oxidative stress and inflammatory parameters***

There was no significant difference in the MDA and SOD concentrations among the 6 groups of the study, but it was significant for the IL-6 level (*P*-value =0.000), Table 3. Table 3 also shows that consumption of different oils (sunflower, mixture, WPH and WPK) significantly decreased the IL-6 level compared to the fructose group (*P*-value =0.003, *P*-value =0.000, *P*-value =0.000 and *P*-value =0.004) and the most reduction was related to the groups who consumed mixture and WPH oils.

***Weight changes***

Figure 1 shows weight changes in different groups during 10 weeks of the study. In Table 2, based on ANOVA analysis, we can see that weight changes during the study were significantly different among the 6 groups of rats (*P*-value =0.005). Based on *post-hoc* analysis, fructose consumption increased the weight significantly compared to the control group (*P*-value =0.001), but consumption of oils decelerated the weight increment; however, it was not significant.

## Discussion

The present study evaluated the preventive effects of WP oils on the elements and risk factors of metabolic syndrome in rats with induced metabolic syndrome. The main findings of this study were that mixture and WPK oils improve lipid profiles, glycemic indices, and inflammation in rats with induced metabolic syndrome. The hypotriglyceridemic and anti-inflammatory effects of WPH oil were also revealed. The hypolipidemic effects of WPK oil, WPH oil, and a mixture of the two can be attributed to the fatty acid composition and biochemical elements of the oils.

In this study, total cholesterol and LDL levels in the WPK oil group, total cholesterol in the mixture oil group and LDL level in sunflower oil group were significantly reduced. These results can be attributed to the high phytosterol content in WP and sunflower oils. Phytosterols as an important component of these oils include beta-sitosterol, campesterol, stigmasterol, and sitostanol among others. These herbal sterols can manage cholesterol absorption and reduce LDL levels because of their similar structures and biological functions with cholesterol. (17, 21). They also empower the ABC transporter function (ATP binding cassette transporter), which can pump cholesterol from enterocytes to the lumen of the small intestine (22). By reducing cholesterol levels, phytosterols can reduce arthrosclerosis risk and inflammatory processes (23, 24). Farquhar *et al.* showed that beta-sitosterol supplementation in individuals with cardiac disease causes reductions in serum cholesterol, beta lipoproteins, and total lipids (25). 

Based on the WHO and NCEP ATP III definitions, hypertriglyceridemia is one component of metabolic syndrome that can contribute to atherogenesis. Lipoproteins, which include triglycerides, can cause atherogenesis by causing disturbances in vasodilation, increasing pre-inflammatory cytokine production, increasing inflammatory responses, and activating monocytes (26, 27). As the current study showed, the triglyceride levels were reduced in sunflower and all 3 WP oil groups in comparison with the fructose group; this result in WP oil groups may be due to the high content of the mono-unsaturated fatty acid, oleic acid. Increasing the oxidation of fatty acids through the activation of peroxisome proliferator-activated receptor α (PPAR α) is a mechanism through which oleic acid can reduce triglycerides in the liver (28). As most serum triglycerides are transferred by VLDL, the production of VLDL-triglycerides in the liver and removing triglycerides from the circulation are two key factors affecting triglyceride concentrations. Two probable hypotriglyceridemia mechanisms by MUFA include changing the composition of VLDL and proteins and enzymes expression which involved in intravascular metabolism and catabolism of VLDL, both of which can reduce plasma triglyceride levels. Dietary fatty acids are important factors in converting VLDL into other lipoproteins and triglyceride metabolisms by changing the combination of VLDL-triglyceride fatty acids (29, 30). On the other hand, a 1999 study by Etherton *et al* . showed that a diet high in mono-unsaturated fatty acids has reductive effects on triglycerides, total cholesterol, and LDL in comparison with a typical American diet. Their results confirm the findings of the current study (31). According to our results (Table 1) sunflower oil has 61 percent linoleic acid and studies showed that PUFA (poly unsaturated fatty acid) can down-regulate SREBP-1 (sterol regulatory element binding proteins) and, thereby, synthesis of triglyceride (32).

HDL is an important element of metabolic syndrome in lipoprotein metabolism and can be a powerful predictive factor in cardiovascular diseases (33, 34). However, the current study revealed a reduction in all components of lipid profiles (such as HDL) in the WPK oil group. WPK oil has about 35% PUFA fatty acid; thus, reduced HDL levels can be a result of the PUFA content in this oil (17). Mattson and Grundy also found a significant reduction in HDL levels in safflower oil receivers and attributed it to the high level of PUFA (35). However, the current study did not show any effects of other oils on HDL, because they all had a lower PUFA content in them. In contrast, Saeb *et al.* showed in their study that a diet with a 10-20% concentration of WPK oil caused reductions in total cholesterol, triglycerides, and LDL in female rabbits; however, unlike the current study, they showed an increased level of HDL. This difference in results can be attributed to different diets and animal models (36).

One impressive result of this study is the reduction in interleukin-6 levels in sunflower and all WP oil groups. In 2011, Lira *et al.* showed anti-inflammatory effects of tocopherol by reducing IL-6 and based on Table 1 sunflower oil has favorable values of total tocopherol that can explain it ‘ s effect on IL-6 reduction (37).

 In 2001, Moreno *et al.* reported reductions in arachidonic acid release and prostaglandin E2 production in the macrophages of rats fed high amounts of oleic acid; these results confirm the anti-inflammatory effects of oleic acid (38). In another study the anti-inflammatory effect of olive oil was evaluated in individuals with cardiovascular disease. The results showed that olive oil can reduce CRP and IL-6 levels (39). Inflammatory factors can motivate greater production and cascading activities in beta pancreas cells, which can then increase the death of beta pancreas cells and inhibit insulin secretion. Therefore, a reduction in the viscosity of inflammatory cytokines in plasma can have medical effects on beta pancreas and diabetic cell disorders (40, 41). In the current study, the consumption of WPK oil increased insulin secretion by reducing IL-6 levels in comparison with the fructose group. These effects were not seen in the rats that consumed the mixture and hull oils of WP since tochopherols have anti-inflammatory properties 

This study also showed that WPK and mixture oils reduced FBG levels and insulin resistance indices. This study and other evaluations have shown that oleic acid 18-1 (n-9) is the main fatty acid of WP oil (hull and kernel) (Table 1), (17, 42), and it has a suitable effect on glycemic indices based on observations of the consumption of a diet high in this fatty acid. Previous studies have shown that oleic acid can reduce hyperglycemia by improving the secretory function of beta pancreas cells, improving semi-glucagon peptide secretion (GLP-1), and combatting the destructive effects of high blood glucose (43-45). WP oil is a mixture of fatty acids with a complex of bioactive components, but the exact mechanism with which WP oil can create metabolic changes in the body is not completely clear. It is reported that a diet containing oleic acid can improve plasma glucose and insulin levels and preserve beta pancreas cells in obese rats (46). In 2011, Bermudez *et al.* reported the improving effect of olive oil on insulin secretion and sensitivity (47).

**Table1:** Chemical composition of wild pistachio kernel (WPK), wild pistachio hull (WPH) and Sunflower oils.

Parameters	WPK oil	WPH oil	Sunflower oil
Fatty acids (percent)			
16:0 18:1 18:2 18:3 Others	0.03 51.17 32.85 0.63 5.32	23.74 54.33 5.84 0.7 15.39	7.39 25 63.2 0.2 4.21
Sterols (mg/kg)			
B-sitosterol Campesterol Others Total	84 4 12 3811.12	82.44 5.68 11.88 1569.47	61.11 8.4 30.49 2700
Total tocopherol (mg/kg)	350.15	1070.49	582
Total phenol (mg/kg)	4.25	89.5	1.2
Acid value (mg KOH/g oil)	0.9	6.78	8
Peroxide value (mequiv O2/kg oil)	0.8	4.2	0.7

**Table 2:** Weight, lipid profile and glycemic indices of each group fed with different oils

** Groups**	**Control** **(mean±SD)**	**Fructose** **(mean±SD)**	**Fructose +** **Sunflower oil** **(mean±SD)**	**Fructose +** **Mixed oil** **(mean±SD)**	**Fructose +** ** Hull oil** **(mean±SD)**	**Fructose +** **Kernel oil ** **(mean±SD)**	***P*** **-value**
**Variables**							
**TG**(mg/dl)	62.63±8.09	107.20±14.14^a^	48.18±11.23^a^^b^	43.36±11.36^a^^b^	55.10±10.76^b^	47.27±11.23^a^^b^	<.001
**T-Chol **(mg/dl)	51.27±7.19	71.40±7.54^a^	61.09±8.37^a^	59.54±8.93^b^	64.30±8.99^a^	57.81±4.62^b^	<.001
**LDL**(mg/dl)	6.81±1.07	16.30±3.74^a^	12.00±2.28^a^^b^	13.63±2.01^a^	13.60±1.57^a^	12.18±2.31^a^^b^	<.001
**HDL**(mg/dl)	35.36±7.32	47.40±4.24^a^	42.45±4.10^a^	43.27±3.52^a^	46.00±5.88^a^	40.27±3.90^b^	<.001
**FBG**(mg/dl)	114.45±28.42	178.00±17.32^a^	174.72±38.38^a^	120.45±14.09^b^^c^	150.30±27.60^a^	123.45±24.88^b^^c^	<.001
**Insulin**(µIU/ml)	1.90±0.24	1.59±0.17a	1.47±0.18^a^	1.78±0.18^c^	1.63±0.22^a^	1.85±0.18^b^^c^	<.001
**HOMA-IR**	0.53±0.13	0.7±0.12^a^	0.64±0.19	0.53±0.10^b^	0.60±0.13	0.56±0.11^b^	.04
**Weight changes **(gr)	82.72±12.74	121.30±20.36^a^	106.45±16.57	104.72±20.19	107.70±32.15	106.54±18.88	.005

* *P*<0.05 considered significant.

a - significant difference compared to the control group.

b - significant difference compared to the fructose group.

c - significant difference compared to the sunflower oil group.

d - significant difference compared to the kernel oil group.

**Table3:** Inflammation and oxidative stress markers of each group fed with different oils.

** Groups**	**Control** **(mean±SD)**	**Fructose** **(mean±SD)**	**Fructose +** **Sunflower oil** **(mean±SD)**	**Fructose +** **Mixed oil** **(mean±SD)**	**Fructose +** ** Hull oil** **(mean±SD)**	**Fructose +** **Kernel oil ** **(mean±SD)**	***P*** **-value**
**Variables**							
**MDA** **(pg/ml)	3.92(3.58-5.25)	4.27(3.75-5.78)	3.83(3.7-4.92)	3.92(3.87-4.85)	4.00(3.76-4.20)	4.08(3.50-4.37)	.86
**SOD**(u/ml)	30.30±1.49	31.82±1.04	32.24±2.81	32.45±1.19	32.29±1.62	31.65±2.44	.11
**IL-6**(pg/ml)	54.97±7.88	69.49±6.39^a^	52.38±5.21^b^	45.47±14.31^b^	43.48±14.37^b^	53.05±7.09^b^	<.001

* *P*<0.05 considered significant.

a - significant difference compared to the control group.

b - significant difference compared to the fructose group.

c - significant difference compared to the sunflower oil group.

d - significant difference compared to the kernel oil group.

** nonparametric data expressed as median (IQR) (IQR= 25^th^ percentile and 75^th^ percentile).

Hyperglycemia increases ROS production in mitochondria by intervening in the respiratory chain, which plays important roles in various metabolic disorders related to the diabetic condition. Today, one of the main strategies for treating diabetes and pancreas secretory disorders is to emphasize reducing oxidative stress (48). As seen in Kaneto *et al.* antioxidant therapy in diabetic mice improved the function of beta pancreas cells and delayed pancreatic disorders of glucose intoxication (49). Therefore, other probable mechanisms of WP oil in reducing hyperglycemia and improving insulin secretion may be attributed to its antioxidant components based on Table 1. Although this study didn’t show any serumic antioxidative effects of the oils but they may improve antioxidant defense system of the cells.

According to previous studies, WP is high in phenols, flavonoids, and tocopherols, and the antioxidant characteristics of WP are attributed to these elements (50, 51). In spite of the current results, previous studies have shown that extracts of WP components improve the oxidative situation; this paradox is attributed to the intervention methods of these studies, which included the usage of hydro-alcoholic extracts of WP (52, 53). Based on such studies, hydro-alcoholic extract is one of the best methods of extracting polyphenolic contents. The method used in the current study was to extract the oil from WP fruit (kernel and external hull) using the cold press method; this could explain the lack of effect that WP oil had on oxidative stress indices in this study because despite high phenolic level that is lower compared to hydro-alcoholic extract form (54, 55). It is possible that more time was required for these effects to be seen. It is worthy to note that this research was a preventative study, and that may have affected the results. 

Weight increment and obesity are mentioned as backgrounds for metabolic syndrome. It is noteworthy that different intervention groups in the current study had significant weight increases during the ten-week period; the groups receiving WP oils had lower increases in weight than the fructose group, however the difference was not significant. The effects of WP oils in this relation may be attributed to the increased lipid metabolism by activation of PPAR-α by oleic acid (28).

In this study, the better effects of WPK oil compared with WPH oil can be explained by the fact that the kernel of WP is surrounded by a hard, wooden shell, and its exposure to oxidation factors such as sunlight, oxygen, and humidity is less; thus, the peroxide value of WPK oil is lower than that of the WPH oil (12). As seen in Table 1, WPH oil, despite having greater antioxidant contents, was probably oxidized before the oil was extracted, and thus it had no primary effects. Mixing WPH oil and WPK oil also partly increase therapeutic effects of WPH oil.

In general, based on the results of the current study, it is proposed that WP kernel oil can be used in treating metabolic syndrome and human studies in the future, in addition long term preventive studies to evaluate antioxidant effects of the oils is recommended. 

Some limitations of this study may have been the lack of blood pressure and waist circumference measurements for the rats.

## Conclusion

Results of the current study showed that consuming WP kernel oil was more effective than other oils (WPH and mixture oils) in preventing hyperglycemia, hypertriglyceridemia, hypercholesterolemia, inflammation and pancreatic secretory disorders; therefore, it may reduce metabolic syndrome risk. In addition it seems that the use of this oil may be effective in treatment of metabolic syndrome that is recommended in future studies.

## References

[1] Grundy SM, Brewer HB, Cleeman JI, Smith SC, Lenfant C (2004). Definition of metabolic syndrome. Arterioscler Thromb Vasc Biol.

[2] Gillingham LG, Harris-Janz S, Jones PJ (2011). Dietary monounsaturated fatty acids are protective against metabolic syndrome and cardiovascular disease risk factors. Lipids.

[3] Alberti KGM, Zimmet P, Shaw J (2005). The metabolic syndrome—a new worldwide definition. Lancet.

[4] Carroll S, Dudfield M (2004). What is the relationship between exercise and metabolic abnormalities?. Sports Med.

[5] Cheal KL, Abbasi F, Lamendola C, McLaughlin T, Reaven GM, Ford ES (2004). Relationship to insulin resistance of the adult treatment panel III diagnostic criteria for identification of the metabolic syndrome. Diabetes.

[6] Fujita K, Nishizawa H, Funahashi T, Shimomura I, Shimabukuro M (2006). Systemic oxidative stress is associated with visceral fat accumulation and the metabolic syndrome. Circ J.

[7] Riccardi G, Rivellese A (2000). Dietary treatment of the metabolic syndrome—the optimal diet. Br J Nutr.

[8] Ford ES, Mokdad AH, Giles WH, Brown DW (2003). The metabolic syndrome and antioxidant concentrations. Diabetes.

[9] Kastorini C-M, Milionis HJ, Esposito K, Giugliano D, Goudevenos JA, Panagiotakos DB (2011). The effect of mediterranean diet on metabolic syndrome and its components: a meta-analysis of 50 studies and 534,906 individuals. J Am Coll Cardiol.

[10] Nejabat M, Negahdarsaber M, Ghahari G (2017). Range of soil and climate characteristics appropriate for Pistacia atlantica forest development an d rehabilitation (case study: Fars province, Iran). JWLD.

[11] Pourreza M, Shaw JD, Zangeneh H (2008). Sustainability of wild pistachio (Pistacia atlantica Desf) in Zagros forests, Iran. Forest Ecology and Management.

[12] Farhoosh R, Khodaparast MHH, Sharif A (2009). Bene hull oil as a highly stable and antioxidative vegetable oil. Eur J Lipid Sci.

[13] Memariani Z, Sharifzadeh M, Bozorgi M, Hajimahmoodi M, Farzaei MH, Gholami M (2017). Protective effect of essential oil of Pistacia atlantica Desf On peptic ulcer: role of α-pinene. J Tradit Chin Med.

[14] Hashemnia M, Nikousefat Z, Yazdani-Rostam M (2015). Antidiabetic effect of Pistacia atlantica and Amygdalus scoparia in streptozotocin-induced diabetic mice. Comp Clin Pathol.

[15] Tanideh N, Masoumi S, Hosseinzadeh M, Safarpour AR, Erjaee H, Koohi-Hosseinabadi O (2014). Healing effect of Pistacia atlantica fruit oil extract in acetic Acid-induced colitis in rats. Iran J Med Sci.

[16] Arami S, Mojaddadi M, Pourabbas R, Chitsaz M, Delazar A, Mobayen H (2015). The effect of Pistacia atlantica Var mutica mouthwash on dental plaque bacteria and subgingival microorganisms: a randomized and controlled triple-blind study. Drug Res.

[17] Saber-Tehrani M, Givianrad M, Aberoomand-Azar P, Waqif-Husain S, Jafari Mohammadi S (2012). Chemical composition of Iran’s Pistacia atlantica cold-pressed oil. J Chem.

[18] Barbosa C, Albuquerque E, Faria E, Oliveira H, Castilho L (2007). Opposite lipemic response of Wistar rats and C57BL/6 mice to dietary glucose or fructose supplementation. Braz J Med Biol Res.

[19] Zal F, Mostafavi-Pour Z, Vessal M (2007). Comparison of the effects of vitamin e and/or quercetin in attenuating chronic cyclosporine a-induced nephrotoxicity in male rats. Clin Exp Pharmacol Physiol.

[20] Matthews D, Hosker J, Rudenski A, Naylor B, Treacher D, Turner R (1985). Homeostasis model assessment: insulin resistance and β-cell function from fasting plasma glucose and insulin concentrations in man. Diabetologia.

[21] Rozner S, Garti N (2006). The activity and absorption relationship of cholesterol and phytosterols. Colloids Surf A.

[22] Włodarek D (2007). The mechanisms of blood LDL-cholesterol lowering by phytosterols-a review. Rocz Panstw Zakl Hig.

[23] Berger A, Jones PJ, Abumweis SS (2004). Plant sterols: factors affecting their efficacy and safety as functional food ingredients. Lipids Health Dis.

[24] Law M (2000). Plant sterol and stanol margarines and health. BMJ.

[25] Farquhar JW, Smith RE, Dempsey ME (1956). The effect of beta sitosterol on the serum lipids of young men with arteriosclerotic heart disease. Circulation.

[26] Alberti KGMM, Zimmet P, Shaw J (2006). Metabolic syndrome—a new world-wide definition A consensus statement from the international diabetes federation. Diabet Med.

[27] Ginsberg HN (2002). New perspectives on atherogenesis. Circulation.

[28] Assy N, Nassar F, Nasser G, Grosovski M (2009). Olive oil consumption and non-alcoholic fatty liver disease. World J Gastroenterol.

[29] Maeda H, Hosokawa M, Sashima T, Miyashita K (2007). Dietary combination of fucoxanthin and fish oil attenuates the weight gain of white adipose tissue and decreases blood glucose in obese/diabetic KK-Ay mice. J Agric Food Chem.

[30] Mcnamara DJ (1992). Dietary fatty acids, lipoproteins, and cardiovascular disease. Adv Food Nutr Res.

[31] Kris-Etherton PM, Pearson TA, Wan Y, Hargrove RL, Moriarty K, Fishell V (1999). High–monounsaturated fatty acid diets lower both plasma cholesterol and triacylglycerol concentrations. Am J Clin Nutr.

[32] Clarke SD (2001). Polyunsaturated fatty acid regulation of gene transcription: a molecular mechanism to improve the metabolic syndrome. J Nutr.

[33] Karadag MK, Akbulut M (2009). Low HDL levels as the most common metabolic syndrome risk factor in heart failure. Int Heart J.

[34] Freitas H, Barbosa E, Rosa F, Lima A, Mansur A (2009). Association of HDL cholesterol and triglycerides with mortality in patients with heart failure. Braz J Med Biol Res.

[35] Mattson FH, Grundy SM (1985). Comparison of effects of dietary saturated, monounsaturated, and polyunsaturated fatty acids on plasma lipids and lipoproteins in man. J Lipid Res.

[36] Saeb M, Nazifi S, Mirzaei A (2004). Studies on the effects of turpentine oil on the serum concentration of lipids and lipoproteins of female rabbits. SSU J.

[37] Lira FS, Rosa JC, Cunha CA, Ribeiro EB, do Nascimento CO, Oyama LM (2011). Supplementing alpha-tocopherol (vitamin E) and vitamin D3 in high fat diet decrease IL-6 production in murine epididymal adipose tissue and 3T3-L1 adipocytes following LPS stimulation. Lipids Health Dis.

[38] Moreno JJ, Carbonell T, Sanchez T, Miret S, Mitjavila MT (2001). Olive oil decreases both oxidative stress and the production of arachidonic acid metabolites by the prostaglandin G/H synthase pathway in rat macrophages. J Nutr.

[39] Fitó M, Cladellas M, De la Torre R, Marti J, Munoz D, Schröder H (2008). Anti-inflammatory effect of virgin olive oil in stable coronary disease patients: a randomized, crossover, controlled trial. Eur J Clin Nutr.

[40] Igoillo-Esteve M, Marselli L, Cunha D, Ladrière L, Ortis F, Grieco FA (2010). Palmitate induces a pro-inflammatory response in human pancreatic islets that mimics CCL2 expression by beta cells in type 2 diabetes. Diabetologia.

[41] Eizirik DL, Colli ML, Ortis F (2009). The role of inflammation in insulitis and β-cell loss in type 1 diabetes. Nat Rev Endocrinol.

[42] Toul F, Belyagoubi-Benhammou N, Zitouni A, Atik-Bekkara F (2017). Antioxidant activity and phenolic profile of different organs of Pistacia atlantica Desf subsp atlantica from Algeria. Nat Prod Res.

[43] Maedler K, Oberholzer J, Bucher P, Spinas GA, Donath MY (2003). Monounsaturated fatty acids prevent the deleterious effects of palmitate and high glucose on human pancreatic β-cell turnover and function. Diabetes.

[44] Garg A (1994). High monounsaturated fat diet for diabetic patients: Is it time to change the current dietary recommendations?. Diabetes Care.

[45] Rocca AS, LaGreca J, Kalitsky J, Brubaker PL (2001). Monounsaturated fatty acid diets improve glycemic tolerance through increased secretion of glucagon-like peptide-1. Endocrinology.

[46] Sato K, Arai H, Mizuno A, Fukaya M, Sato T, Koganei M (2007). Dietary palatinose and oleic acid ameliorate disorders of glucose and lipid metabolism in Zucker fatty rats. J Nutr.

[47] Bermudez B, Lopez S, Ortega A, M Varela L, M Pacheco Y, Abia R (2011). Oleic acid in olive oil: from a metabolic framework toward a clinical perspective. Curr Pharm Des.

[48] Rolo AP, Palmeira CM (2006). Diabetes and mitochondrial function: role of hyperglycemia and oxidative stress. Toxicol Appl Pharmacol.

[49] Kaneto H, Kajimoto Y, Miyagawa J-i, Matsuoka T-a, Fujitani Y, Umayahara Y (1999). Beneficial effects of antioxidants in diabetes: possible protection of pancreatic beta-cells against glucose toxicity. Diabetes.

[50] Belyagoubi-Benhammou N, Belyagoubi L, El Zerey-Belaskri A, Atik Bekkara F (2014). In vitro antioxidant properties of flavonoid fractions from Pistacia atlantica Desf subsp atlantica fruit using five techniques. J Mater Environ Sci.

[51] Farhoosh R, Tavakoli J, Khodaparast MHH (2008). Chemical composition and oxidative stability of kernel oils from two current subspecies of Pistacia atlantica in Iran. J Am Oil Chem’ Soc.

[52] Rashidi S, Askari N, Abbasnejad M (2014). Anxiolytic-like effect of Pistacia atlantica fruit in intact and gonadectomized rats subjected to chronic stress. JOHE.

[53] Farahpour MR, Mirzakhani N, Doostmohammadi J, Ebrahimzadeh M (2015). Hydroethanolic Pistacia atlantica hulls extract improved wound healing process; evidence for mast cells infiltration, angiogenesis and RNA stability. Int J Surg.

[54] Harborne A (1998). Phytochemical methods a guide to modern techniques of plant analysis.

[55] Dai J, Mumper RJ (2010). Plant phenolics: extraction, analysis and their antioxidant and anticancer properties. Molecules.

